# Stochastic ***θ***-Methods for a Class of Jump-Diffusion Stochastic Pantograph Equations with Random Magnitude

**DOI:** 10.1155/2014/589167

**Published:** 2014-02-06

**Authors:** Hua Yang, Feng Jiang

**Affiliations:** ^1^School of Automation, Huazhong University of Science and Technology, Wuhan 430074, China; ^2^School of Mathematics and Computer Science, Wuhan Polytechnic University, Wuhan 430023, China; ^3^School of Statistics and Mathematics, Zhongnan University of Economics and Law, Wuhan 430073, China

## Abstract

This paper is concerned with the convergence of stochastic *θ*-methods for stochastic pantograph equations with Poisson-driven jumps of random magnitude. The strong order of the convergence of the numerical method is given, and the convergence of the numerical method is obtained. Some earlier results are generalized and improved.

## 1. Introduction

Recently, the study of stochastic pantograph equations (SPEs) has many results [1–3]. SPEs have been extensively applied in many fields such as finance, control, and engineering. However, in general, SPEs have no explicit solutions, and the study of numerical solutions of SPEs has received a great deal of attention. Fan et al. [4] investigate the *α*th moment asymptotical stability of the analytic solution and the numerical methods for the stochastic pantograph equation by using the Razumikhin technique. Baker and Buckwar [5] gave strong approximations to the solution obtained by a continuous extension of the *θ*-Euler scheme and proved that the numerical solution produced by the continuous *θ*-method converges to the true solution with order 1/2. Fan et al. [6] investigated the existence and uniqueness of the solutions and convergence of semi-implicit Euler methods for stochastic pantograph equations under the local Lipschitz condition and the linear growth condition. Li et al. [7] investigated the convergence of the Euler method of the stochastic pantograph equations with Markovian switching under the weaker conditions. Reference [8] studied convergence and stability of numerical methods of stochastic pantograph differential equations.

In practice, stochastic differential equations with jump and numerical methods are also discussed extensively. In [9–13] strong convergence and mean-square stability properties were analysed in the case of Poisson-driven jumps of deterministic magnitude. References [14, 15] discussed the numerical methods of stochastic differential equations with random jump magnitudes. Motivated by the papers above, in this paper, we focus on stochastic pantograph equations with random jump magnitudes. SPEs with random jump magnitudes may be regarded as an extension of stochastic pantograph equations. Jump models arise in many other application areas and have proved successful at describing unexpected, abrupt changes of state [16–18]. Typically, these models do not admit analytical solutions and hence must be simulated numerically. Similar to stochastic differential equations [19–21], explicit solutions can hardly be obtained for SPEs with random jump magnitudes. Thus, appropriate numerical approximation schemes such as the Euler (or Euler-Maruyama) are needed to apply them in practice or to study their properties.

The paper is organised as follows. In Section 2, we introduce the SPEs with random jump magnitudes and define stochastic *θ*-methods of (1). The main result of the paper is rather technical, so we present several lemmas in Section 3 and then complete the proof in Section 4.

## 2. Preliminaries

Throughout this paper, we let (*Ω*, *ℱ*, {*ℱ*_*t*_}_*t*≥0_, *P*) be a complete probability space with a filtration {*ℱ*_*t*_}_*t*≥0_ satisfying the usual conditions (i.e., it is increasing and right continuous while *ℱ*_0_ contains all *P*-null sets). Let |·| be the Euclidean norm in *ℛ* ^*n*^. Let *x*_0_ be *ℱ*_0_-measurable and right-continuous, and *E* | *x*_0_|^2^ < *∞*. Let *w*(*t*) = (*w*_*t*_^1^,…,*w*_*t*_^*m*^)^*T*^ be a *m*-dimensional Brownian motion defined on the probability space.

Consider a class of jump-diffusion stochastic pantograph equations with random magnitude of the form

(1) dx(t)=f(x(t−),x(μt−))dt+g(x(t−),x(μt−))dw(t)+h(x(t−),x(μt−),γN(t−)+1)dN(t),

on 0 ≤ *t* ≤ *T* with the initial value *x*(0^−^) = *x*_0_ and 0 < *μ* < 1, where *N*(*t*) is a Poisson process with mean *λt*; *x*(*t* ^−^)∶ = lim⁡_*s*→*t*^−^_ *x*(*s*); and *γ*_*i*_, *i* = 1,2,… are independent, identically distributed random variables representing magnitudes for each jump.

Throughout, we assume that the jump magnitudes have bounded moments; that is, for some *q* ≥ 1, there is a constant *B* = *B*_*q*_ such that

(2) $$\begin{array}{c} E\left({\left|\gamma_{i}\right|}^{2q}\right)\leq B. \end{array}$$

We further employ the following assumptions.

**Assumption 1**

The functions *f*, *g*, and *h* satisfy the global Lipschitz condition, that is, for each *k* = 1,2, 3, there is a positive constant *K*_1_ such that

(3) |f(x1,x2)−f(y1,y2)|2∨|g(x1,x2)−f(y1,y2)|2  ≤K1(|x1−y1|2+|x2−y2|2),|h(x1,x2,x3)−h(y1,y2,y3)|2  ≤K1(|x1−y1|2+|x2−y2|2+|x3−y3|2),

where *x*_*k*_, *y*_*k*_ ∈ *ℜ* ^*n*^.

**Assumption 2 (linear growth condition)**

There is a positive constant *K*_2_ such that for all *t* ∈ [0, *T*]

(4) $$\begin{array}{c} {\left|f\left(x_{1},x_{2}\right)\right|}^{2}\vee {\left|g\left(x_{1},x_{2}\right)\right|}^{2}\leq K_{2}\left(1+{\left|x_{1}\right|}^{2}+{\left|x_{2}\right|}^{2}\right),\\ {\left|h\left(x_{1},x_{2},x_{3}\right)\right|}^{2}\leq K_{2}\left(1+{\left|x_{1}\right|}^{2}+{\left|x_{2}\right|}^{2}+{\left|x_{3}\right|}^{2}\right), \end{array}$$

for all *x*_1_, *x*_2_, *x*_3_ ∈ *ℜ* ^*n*^.

In fact, the global Lipschitz condition (3) implies the linear growth condition (4). Under these conditions, it can be shown similarly as in [20] that (1) has a unique solution with all moments bounded.

We note for later reference that (1) involves the jump process

(5) $$\begin{array}{c} \gamma \left(t\right):=\gamma_{N(t^{-})+1}=\overset{\infty }{\underset{j=0}{\sum }}\gamma_{j+1}1_{\left[\tau_{j},\tau_{j+1}\right)}\left(t\right), \end{array}$$

where *τ*_0_ = 0 and *τ*_*j*_, *j* = 1,2,… are the jump times.

One generalisation of stochastic *θ*-Euler methods [6, 21] to system (1) has the form *y*_0_ = *x*(0) and

(6) yk+1=yk+(1−θ)f(yk,y[μk])h+θf(yk+1,y[μ(k+1)])h+g(yk,y[μk])Δwk+h(yk,y[μk],γN(tk)+1)ΔNk,

where *θ* ∈ [0,1]. Here *h* ∈ (0,1) is a step size, which satisfies *M* = *T*/*h* for some positive integer *M*, *t*_*k*_ = *kh*  (*k* = 0,1,…, *M*). *y*_*k*_ ≈ *x*(*t*_*k*_), Δ*w*_*k*_ = *w*(*t*_*k*+1_) − *w*(*t*_*k*_), and Δ*N*_*k*_ = *N*(*t*_*k*+1_) − *N*(*t*_*k*_) are the Brownian and Poisson increments, respectively.

For *t* ∈ [*t*_*k*_, *t*_*k*+1_], we define

(7) y(t)=yk+(1−θ)f(yk,y[μk])(t−tk)+θf(yk+1,y[μ(k+1)])(t−tk)+g(yk,y[μk])(w(t)−w(tk))+h(yk,y[μk],γN(tk)+1)(N(t)−N(tk))

and denote

(8) $$\begin{array}{c} z_{1}\left(t\right)=\overset{\infty }{\underset{j=0}{\sum }}y_{j}1_{\left[jh,(j+1)h\right)}\left(t\right),\\ z_{2}\left(t\right)=\overset{\infty }{\underset{j=0}{\sum }}y_{j+1}1_{\left[jh,(j+1)h\right)}\left(t\right),\\ {\bar{z}}_{1}\left(t\right)=\overset{\infty }{\underset{j=0}{\sum }}y_{\left[\mu k\right]}1_{\left[jh,(j+1)h\right)}\left(t\right),\\ {\bar{z}}_{2}\left(t\right)=\overset{\infty }{\underset{j=0}{\sum }}y_{\left[\mu (k+1)\right]}1_{\left[jh,(j+1)h\right)}\left(t\right),\\ \bar{\gamma }\left(t\right)=\overset{\infty }{\underset{j=0}{\sum }}\gamma \left(t_{j}\right)1_{\left[jh,(j+1)h\right)}\left(t\right). \end{array}$$

Then, we define the continuous-time approximation

(9) y(t)=y0+∫0t[(1−θ)f(z1(s),z¯1(s))+θf(z2(s),z¯2(s))]ds +∫0tg(z1(s),z¯1(s))dw(s) +∫0th(z1(s),z¯1(s),γ¯(s))dN(s),

which interpolates the discrete numerical approximation (6). So a convergence result for *y*(*t*) immediately provides a result for *y*_*k*_.

## 3. Lemmas

Throughout our analysis, *C*_*i*_, *D*_*i*_, *i* = 1,2,… denote generic constants, independent of *h*. The main theorem of the paper is rather technical. We will present a number of useful lemmas in the section and then complete the proof in Section 4.

**Lemma 3**

Under Assumption 2, there exists 0 < h* < 1 such that for all 0 < *h* ≤ h*,

(10) $$\begin{array}{c} E{\left|y_{k}\right|}^{2}\leq C_{1},\;for\;\;k=1,\ldots ,M. \end{array}$$

**Proof**

From (6), we have

(11) E|yk+1|2≤4E|yk|2+4E|(1−θ)f(yk,y[μk])h    +θf(yk+1,y[μ(k+1)])h|2+4E|g(yk,y[μk])Δwk|2+4E|h(yk,y[μk],γN(tk)+1)ΔNk|2.

Note that 2*αβ* ≤ |*α*|^2^ + |*β*|^2^. Now, using Assumption 2,

(12) E|(1−θ)f(yk,y[μk])h+θf(yk+1,y[μ(k+1)])h|2≤(1−θ)2h2E|f(yk,y[μk])|2+θ2h2|f(yk+1,y[μ(k+1)])|2 +(1−θ)θh2E[|f(yk,y[μk])|2+|f(yk+1,y[μ(k+1)])|2]≤K2[(1−θ)2h2+θ2h2+(1−θ)θh2] +K2[(1−θ)2h2+(1−θ)θh2]E|yk|2 +K2[θ2h2+(1−θ)θh2]E|yk+1|2 +K2[(1−θ)2h2+(1−θ)θh2]E|y[μk]|2 +K2[θ2h2+(1−θ)θh2]E|y[μ(k+1)]|2.

Using *E* | Δ*w*_*k*_|^2^ = *mh* and Assumption 2, we have

(13) $$\begin{array}{c} E{\left|g\left(y_{k},y_{\left[\mu k\right]}\right)\Delta w_{k}\right|}^{2}=mhK_{2}\left(1+E{\left|y_{k}\right|}^{2}+E{\left|y_{\left[\mu k\right]}\right|}^{2}\right). \end{array}$$

For the jump, we convert to the compensated Poisson increment $\Delta {\tilde{N}}_{k}∶=\Delta N_{k}-\lambda h$ with $E(\Delta {\tilde{N}}_{k})=0$ and $E{\left(\Delta {\tilde{N}}_{k}\right)}^{2}=\lambda h$ and Assumption 2. We then obtain

(14) E|h(yk,y[μk],γN(tk)+1)ΔN(s)|2 =E|h(yk,y[μk],γN(tk)+1)(ΔN~(s)+λh)|2 =λh(1+λh)K2(1+E|yk|2+E|y[μk]|2+E|γN(tk)+1|2).

Combining (13), (14), and (15) with (12) yields

(15) E|yk+1|2≤A1(h)+A2(h)E|yk|2+A3(h)E|y(k+1)|2+A4(h)E|y[μk]|2+A5(h)E|y[μ(k+1)]|2+4K2λh(1+λh)E|γN(tk)+1|2≤A1(h)+A3(h)E|y(k+1)|2+4K2λh(1+λh)E|γN(tk)+1|2+(A2(h)+A4(h)+A5(h))max⁡[μk]≤i≤kE|yi|2,

where *A*_*i*_(*h*), *i* = 1,…, 5 is a constant dependent on *h* and *A*_3_(*h*) = 4*K*_2_ *h* ^2^(*θ* ^2^ + (1 − *θ*)*θ*).

Now choosing *h* sufficiently small such that 1 − *A*_3_(*h*) ≥ 1/2 and noting that (2) implies that each *E* | *γ*(*t*_*j*_)|^2^ ≤ *B*_1_, we obtain

(16) E|yk+1|2≤2A1(h)+8K2λh(1+λh)B1+2(A2(h)+A4(h)+A5(h))max⁡[μk]≤i≤kE|yi|2.

The result then follows from an application of the discrete Gronwall inequality. The proof is complete.

**Lemma 4**

Under Assumption 2, there exists h* > 0 such that, for all 0 < *h* ≤ h*,

(17) $$\begin{array}{c} E{\left|y\left(t\right)-z_{1}\left(t\right)\right|}^{2}\leq C_{3}h,\;for\;\;t\in \left[0,T\right], \end{array}$$

(18) $$\begin{array}{c} E{\left|y\left(t\right)-z_{2}\left(t\right)\right|}^{2}\leq C_{4}h,\;for\;\;t\in \left[0,T\right]. \end{array}$$

**Proof**

Consider *t* ∈ [*kh*, (*k* + 1)*h*]⊆[0, *T*]. In this interval we have

(19) y(t)−z1(t)=y(t)−yk=(1−θ)f(yk,y[μk])(t−tk)+θf(yk+1,y[μ(k+1)])(t−tk)+g(yk,y[μk])(w(t)−w(tk))+h(yk,y[μk],γN(tk)+1)(N(t)−N(tk)).

Thus,

(20) E|y(t)−z1(t)|2  ≤3E|(1−θ)f(yk,y[μk])(t−tn)      +θf(yk+1,y[μ(k+1)])(t−tn)|2   +3E|g(yk,y[μk])(w(t)−w(tk))|2   +3E|h(yk,y[μk],γN(tk)+1)(N(t)−N(tk))|2.

Thus, by virtue of (12)–(14) and Lemma 3, we have

(21) E|y(t)−z1(t)|2 ≤3mh2K2(1+2C1)+3λh(1+λh)  ×K2(1+2C1+B1)+15K2h2 ≤C3h,

where *C*_3_ = 3*mK*_2_(1 + 2*C*_1_) + 3*λ*(1 + *λ*)*K*_2_(1 + 2*C*_1_ + *B*_1_) + 15*K*_2_.

In a similar way we obtain (18). The proof is complete.

**Lemma 5**

Under Assumption 2, there exists h* > 0 such that, for all 0 < *h* ≤ h*,

(22) $$\begin{array}{c} E{\left|y\left(\mu t\right)-{\bar{z}}_{1}\left(t\right)\right|}^{2}\leq C_{5}h,\;for\;\;t\in \left[0,T\right],\\ E{\left|y\left(\mu t\right)-{\bar{z}}_{2}\left(t\right)\right|}^{2}\leq C_{6}h\;for\;\;t\in \left[0,T\right]. \end{array}$$

**Proof**

Consider *t* ∈ [*kh*, (*k* + 1)*h*]⊆[0, *T*]. By (9), we have

(23) y(μt)−z¯1(t)=y(μt)−y[μk]=y(μt)−y([μk]h)=∫[μk]hμt[(1−θ)f(z1(s),z¯1(s))+θf(z2(s),z¯2(s))]ds +∫[μk]hμtg(z1(s),z¯1(s))dw(s) +∫[μk]hμth(z1(s),z¯1(s),γ¯(s))dN(s).

Thus,

(24) |y(μt)−z¯1(t)|2≤3|∫[μk]hμt[(1−θ)f(z1(s),z¯1(s))+θf(z2(s),z¯2(s))]ds|2 +3|∫[μk]hμtg(z1(s),z¯1(s))dw(s)|2 +3|∫[μk]hμth(z1(s),z¯1(s),γ¯(s))dN(s)|2≤3|∫[μk]hμt[(1−θ)f(z1(s),z¯1(s))+θf(z2(s),z¯2(s))]ds|2 +3|∫[μk]hμtg(z1(s),z¯1(s))dw(s)|2 +6|∫[μk]hμth(z1(s),z¯1(s),γ¯(s))dN~(s)|2 +6λ2|∫[μk]hμth(z1(s),z¯1(s),γ¯(s))ds|2.

Therefore, in view of the Hölder inequality and *μt* − [*μk*]*h* ≤ 2*h*, we have

(25) E|y(μt)−z¯1(t)|2≤12hE∫[μk]hμt[|f(z1(s),z¯1(s))|2+|f(z2(s),z¯2(s))|2]ds +12E|∫[μk]hμtg(z1(s),z¯1(s))dw(s)|2 +24E|∫[μk]hμth(z1(s),z¯1(s),γ¯(s))dN~(s)|2 +12hλ2E∫[μk]hμt|h(z1(s),z¯1(s),γ¯(s))|2ds.

Then, applying Itô and martingale isometries and Assumption 2, we have

(26) E|y(μt)−z¯1(t)|2≤12hK2∫[μk]hμt[2+E|z1(s)|2+E|z¯1(s)|2       +E|z2(s)|2+E|z¯2(s)|2]ds +12K2∫[μk]hμt(1+E|z1(s)|2+E|z¯1(s)|2)ds +24λK2∫[μk]hμt(1+E|z1(s)|2+E|z¯1(s)|2+E|γ¯(s)|2)ds +12hλ2K2 ×∫[μk]hμt(1+E|z1(s)|2+E|z¯1(s)|2+E|γ¯(s)|2)ds.

Now, note that (2) implies that each *E* | *γ*(*t*_*j*_)|^2^ ≤ *B*_1_, on [*kh*, (*k* + 1)*h*], *z*_1_ ≡ *y*_*k*_, *z*_2_ ≡ *y*_*k*+1_, ${\bar{z}}_{1}\equiv y_{k-m}$, ${\bar{z}}_{2}\equiv y_{k-m+1}$, and $\bar{\gamma }\equiv \gamma_{k}$. Hence, applying Lemma 3, we obtain

(27) E|y(μt)−z¯1(t)|2  ≤48K2h2(1+2C1)+24K2h(1+2C1)   +48K2λh(1+3C1)+24K2λ2h2(1+3C1)  ≤C5h,

where *C*_5_ = 72*K*_2_(1 + 2*C*_1_) + 24*K*_2_ *λ*(2 + *λ*)(1 + 3*C*_1_). In the following we consider $E|y(\mu t)-{\bar{z}}_{2}(t){|}^{2}$:

(28) E|y(μt)−z¯2(t)|2  =E|y(μt)−y[μ(k+1)]|2  ≤2E|y(μt)−y[μk]|2+2E|y[μk]−y[μ(k+1)]|2  ≤4C5h.

Let *C*_6_ = 4*C*_5_; the proof is complete.

## 4. Main Results

We can now state and prove our main result of this paper.

**Theorem 6**

Under Assumption 1 for some *q* > 1 and Assumptions 1–2, there exists h* > 0 and *C* = *C*(*q*) such that, for all 0 < *h* < h*,

(29) $$\begin{array}{c} E\left[\underset{t\in \left[0,T\right]}{\sup}{\left|y\left(t\right)-x\left(t\right)\right|}^{2}\right]\leq Ch^{1-(1/q)}. \end{array}$$

**Proof**

The analysis uses ideas from [15], where analogous results are derived in the stochastic differential equations. By construction, we have

(30) y(t)−x(t)=∫0t(1−θ)[f(z1(s),z¯1(s))−f(x(s−),x(μs−))]ds +∫0tθ[f(z2(s),z¯2(s))−f(x(s−),x(μs−))]ds +∫0t[g(z1(s),z¯1(s))−g(x(s−),x(μs−))]dw(s) +∫0t[h(z1(s),z¯1(s),γ¯(s))     −h(z1(s),z¯1(s),γ(s−))]dN(s) +∫0t[h(z1(s),z¯1(s),γ(s−))     −h(x(s−),x(μs−),γ(s−))]dN(s).

Now for any 0 ≤ *t*_1_ ≤ *T* we have

(31) E(sup⁡t∈[0,t1]|y(t)−x(t)|2)=4E(sup⁡t∈[0,t1]|∫0t((1−θ)[f(z1(s),z¯1(s))              −f(x(s−),x(μs−))]           +θ[f(z2(s),z¯2(s))              −f(x(s−),x(μs−))])ds|2) +4E(sup⁡t∈[0,t1]|∫0t[g(z1(s),z¯1(s))           −g(x(s−),x(μs−))]dw(s)|2) +4E(sup⁡t∈[0,t1]|∫0t‍[h(z1(s),z¯1(s),γ¯(s))           −h(z1(s),z¯1(s),γ(s−))]dN(s)|2) +4E(sup⁡t∈[0,t1]|∫0t[h(z1(s),z¯1(s),γ(s−))           −h(x(s−),x(μs−),γ(s−))]dN(s)|2).

By Assumption 1 and Hölder inequality, we have

(32) E(sup⁡t∈[0,t1]|∫0t((1−θ)[f(z1(s),z¯1(s))−f(x(s−),x(μs−))]        +θ[f(z2(s),z¯2(s))          −f(x(s−),x(μs−))])ds|2)≤2E(sup⁡t∈[0,t1]∫0t12ds∫0t1(|f(z1(s),z¯1(s))               −f(x(s−),x(μs−))|2              +|f(z2(s),z¯2(s))                −f(x(s−),x(μs−))|2)ds)≤2TK1∫0t1(E|z1(s)−x(s−)|2+E|z¯1(s)−x(μs−)|2        +E|z2(s)−x(s−)|2+E|z¯2(s)−x(μs−)|2)ds.

By Assumption 1, the Cauchy-Schwarz inequality, and the Doob inequality in the two martingale terms and the martingale isometry,

(33) E(sup⁡t∈[0,t1]|∫0t[g(z1(s),z¯1(s))−g(x(s−),x(μs−))]dw(s)|2) ≤4E∫0t1|g(z1(s),z¯1(s))−g(x(s−),x(μs−))|2ds ≤4K1∫0t1[E|z1(s)−x(s−)|2+E|z¯1(s)−x(μs−)|2]ds,

(34) E(sup⁡t∈[0,t1]|∫0t[h(z1(s),z¯1(s),γ(s−))        −h(x(s−),x(μs−),γ(s−))]dN(s)|2)=E(sup⁡t∈[0,t1]|∫0t[h(z1(s),z¯1(s),γ(s−))         −h(x(s−),x(μs−),γ(s−))]dN~(s)       +λ∫0t[h(z1(s),z¯1(s),γ(s−))           −h(x(s−),x(μs−),γ(s−))]ds|2)≤2E(sup⁡t∈[0,t1]|∫0t[h(z1(s),z¯1(s),γ(s−))          −h(x(s−),x(μs−),γ(s−))]dN~(s)|2) +2λ2E(sup⁡t∈[0,t1]|∫0t[h(z1(s),z¯1(s),γ(s−))            −h(x(s−),x(μs−),γ(s−))]ds|2)≤8E|∫0t1[h(z1(s),z¯1(s),γ(s−))      −h(x(s−),x(μs−),γ(s−))]dN~(s)|2 +2λ2TE(∫0t1|h(z1(s),z¯1(s),γ(s−))          −h(x(s−),x(μs−),γ(s−))|2ds)≤8λE(∫0t1|h(z1(s),z¯1(s),γ(s−))       −h(x(s−),x(μs−),γ(s−))|2ds) +2λ2TE(∫0t1|h(z1(s),z¯1(s),γ(s−))          −h(x(s−),x(μs−),γ(s−))|2ds)≤8λK1∫0t1(E|z1(s)−x(s−)|2+E|z¯1(s)−x(μs−)|2)ds +2λ2TK1∫0t1(E|z1(s)−x(s−)|2+E|z¯1(s)−x(μs−)|2)ds.

We also have

(35) E(sup⁡t∈[0,t1]|∫0t[h(z1(s),z¯1(s),γ¯(s))       −h(z1(s),z¯1(s),γ(s−))]dN(s)|2)=E(sup⁡t∈[0,t1]|∫0t[h(z1(s),z¯1(s),γ¯(s))         −h(z1(s),z¯1(s),γ(s−))]dN~(s)        +λ∫0t[h(z1(s),z¯1(s),γ¯(s))             −h(z1(s),z¯1(s),γ(s−))]ds|2)≤2E(sup⁡t∈[0,t1]|∫0t[h(z1(s),z¯1(s),γ¯(s))          −h(z1(s),z¯1(s),γ(s−))]dN~(s)|2) +2λ2E(sup⁡t∈[0,t1]|∫0t[h(z1(s),z¯1(s),γ¯(s))           −h(z1(s),z¯1(s),γ(s−))]ds|2)≤8E|∫0t1[h(z1(s),z¯1(s),γ¯(s))      −h(z1(s),z¯1(s),γ(s−))]dN~(s)|2 +2λ2TE(∫0t1|h(z1(s),z¯1(s),γ¯(s))          −h(z1(s),z¯1(s),γ(s−))|2ds)≤8λE(∫0t1|h(z1(s),z¯1(s),γ¯(s))       −h(z1(s),z¯1(s),γ(s−))|2ds) +2λ2TE(∫0t1|h(z1(s),z¯1(s),γ¯(s))         −h(z1(s),z¯1(s),γ(s−))|2ds)≤2λ(4+λT)K1E(∫0t1|γ¯(s)−γ(s−)|2ds)≤2λ(4+λT)K1(∑n=0M′−1E(∫tntn+1|γ¯(s)−γ(s−)|2ds)),

where *M*′ is the smallest integer such that *M*′*h* ≥ *t*_1_.

Now the number of nonzero terms in the summation in (34) is a random variable that is not independent of the summands. To obtain a useful bound, we recall the following Young's inequality:

(36) $$\begin{array}{c} ab\leq \frac{q-1}{q}{ɛ}^{1/(q-1)}a^{q/(q-1)}+\frac{1}{qɛ}b^{q}, \end{array}$$

where *a*, *b*, *ɛ* > 0 and 1 < *q* < *∞*.

Hence,

(37) E(∫tntn+1|γ¯(s)−γ(s−)|2ds)  =E(1{ΔNn≥1}∫tntn+1|γ¯(s)−γ(s−)|2ds)  ≤q−1qɛ1/(q−1)E(1{ΔNn≥1})   +1qɛE(∫tntn+1|γ¯(s)−γ(s−)|2ds)q.

Now we can apply the Hölder inequality as follows:

(38) E(∫tntn+1|γ¯(s)−γ(s−)|2ds)q  ≤E[(∫tntn+1ds)q−1∫tntn+1|γ¯(s)−γ(s−)|2qds]  =hq−1E[∫tntn+1|γ¯(s)−γ(s−)|2qds].

Using (37) in (35), we have

(39) E[|γ¯(s)−γ(s−)|2qds]  ≤22q−1(E[|γ¯(s)|2q]+E[|γ(s−)|2q]),

which follows from the Hölder inequality and (2); we yield

(40) E(∫tntn+1|γ¯(s)−γ(s−)|2ds)  ≤q−1qɛ1/(q−1)E(1{ΔNn≥1})   +hq−1qɛE(∫tntn+1|γ¯(s)−γ(s−)|2qds)  ≤q−1qɛ1/(q−1)P(ΔNn≥1)   +hq−1qɛ∫tntn+1E|γ¯(s)−γ(s−)|2qds  ≤q−1qɛ1/(q−1)λh   +hq−1qɛ22q−1∫tntn+1(E|γ¯(s)|2q+E|γ(s−)|2q)ds  ≤q−1qɛ1/(q−1)λh+hqBqɛ22q.

Choosing *ɛ* = *h* ^(*q* − 1)^2^/*q*^, we have

(41) $$\begin{array}{c} E\left(\overset{t_{n+1}}{\underset{t_{n}}{\int }}{\left|\bar{\gamma }\left(s\right)-\gamma \left(s^{-}\right)\right|}^{2}ds\right)\leq \frac{1}{q}\left(\left(q-1\right)\lambda +2^{2q}B\right)h^{2-1/q}. \end{array}$$

Substituting (40) into (34) yields

(42) E(sup⁡t∈[0,t1]|∫0t[h(z1(s),z¯1(s),γ¯(s))       −h(z1(s),z¯1(s),γ(s−))]dN(s)|2)  ≤2λ(4+λT)K1∑n=0M−11q((q−1)λ+22qB)h2−1/q  ≤2λq(4+λT)K1((q−1)λ+22qB)Mh2−1/q  ≤2λq(4+λT)TK1((q−1)λ+22qB)h1−1/q

as *Mh* ≤ *T*.

Now, substituting (31), (32), (33), and (41) into (30) yields

(43) E(sup⁡t∈[0,t1]|y(t)−x(t)|2)≤8λTK1q(4+λT)((q−1)λ+22qB)h1−1/q +8K1(T+2+4λ+λ2T)∫0t1E|z1(s)−x(s−)|2ds +8K1(T+2+4λ+λ2T)∫0t1E|z¯1(s)−x(μs−)|2ds +8TK1∫0t1E|z2(s)−x(s−)|2ds +8TK1∫0t1E|z¯2(s)−x(μs−)|2ds≤8λTK1q(4+λT)((q−1)λ+22qB)h1−1/q +16K1(T+2+4λ+λ2T) ×∫0t1(E|z1(s)−y(s)|2+E|y(s)−x(s−)|2)ds +16K1(T+2+4λ+λ2T) ×∫0t1(E|z¯1(s)−y(s−τ)|2+E|y(s−τ)−x(μs−)|2)ds +16TK1∫0t1(E|z2(s)−y(s)|2+E|y(s)−x(s−)|2)ds +16TK1∫0t1(E|z¯2(s)−y(s−τ)|2         +E|y(s−τ)−x(μs−)|2)ds.

From Lemmas 4 and 5, we have

(44) E(sup⁡t∈[0,t1]|y(t)−x(t)|2)≤8λTK1q(4+λT)((q−1)λ+22qB)h1−1/q +16K1C3(T+2+4λ+λ2T)h +16K1C5(T+2+4λ+λ2T)h +16TK1C4h+16TK1C6h+32K1(2T+2+4λ+λ2T) ×∫0t1Esup⁡t∈[0,s]|y(t)−x(t−)|2ds≤32K1(2T+2+4λ+λ2T) ×∫0t1Esup⁡t∈[0,s]|y(t)−x(t−)|2ds+D1h1−1/q,

where *D*_1_∶ = (8*λTK*_1_/*q*)(4 + *λT*)((*q* − 1)*λ* + 2^2*q*^ *B*) + 16*K*_1_((*C*_3_ + *C*_5_)(*T* + 2 + 4*λ* + *λ* ^2^ *T*) + *TC*_4_ + *TC*_6_).

By the Gronwall inequality, we have

(45) $$\begin{array}{c} E\left(\underset{t\in \left[0,T\right]}{\sup}{\left|y\left(t\right)-x\left(t\right)\right|}^{2}\right)\leq D_{1}h^{1-1/q}e^{32K_{1}(2T+2+4\lambda +\lambda^{2}T)}. \end{array}$$

The proof is complete.

**Remark 7**

Theorem 6 shows that the order of convergence in mean square is close to 1. Moreover, stochastic *θ*-methods give strong convergence rate arbitrarily close to order 1/2 under appropriate moment bounds on the jump magnitude. This problem class is now widely used in mathematical finance.

By Theorem 6, we obtain the following corollaries.

**Corollary 8**

Under Assumption 1,

(46) $$\begin{array}{c} \underset{h\to 0}{\lim}E\left[\underset{t\in \left[0,T\right]}{\sup}{\left|y\left(t\right)-x\left(t\right)\right|}^{2}\right]=0. \end{array}$$

The convergent result can be extended to the case of nonlinear coefficients that are local Lipschitz [6, 7, 12] based on the style of analysis in [22].

**Corollary 9**

Under the local Lipschitz condition and Assumption 2,

(47) $$\begin{array}{c} \underset{h\to 0}{\lim}E\left[\underset{t\in \left[0,T\right]}{\sup}{\left|y\left(t\right)-x\left(t\right)\right|}^{2}\right]=0. \end{array}$$

**Remark 10**

Corollary 9 shows that the numerical solution converges to the true solution. However, the order of the convergence of the numerical method is not given under the local Lipschitz condition. If we remove jump and discuss the system without time lag, our results are reduced to the results derived in [6, 14]. In other words, our results are the generalization of paper [6, 14].
